# XRCC1 Is a Promising Predictive Biomarker and Facilitates Chemo-Resistance in Gallbladder Cancer

**DOI:** 10.3389/fmolb.2020.00070

**Published:** 2020-04-24

**Authors:** Zhengchun Wu, Xiongying Miao, Yuanfang Zhang, Daiqiang Li, Qiong Zou, Yuan Yuan, Rushi Liu, Zhulin Yang

**Affiliations:** ^1^Hunan Provincial Key Laboratory of Hepatobiliary Disease Research, Department of General Surgery, Second Xiangya Hospital, Central South University, Changsha, China; ^2^Immunodiagnostic Reagents Engineering Research Center of Hunan Province, School of medicine, Hunan Normal University, Changsha, China; ^3^Department of Pathology, The Second Xiangya Hospital, Central South University, Changsha, China; ^4^Department of Pathology, The Third Xiangya Hospital, Central South University, Changsha, China

**Keywords:** XRCC1, gallbladder cancer, prognosis, clinicopathological significance, chemo-resistance

## Abstract

Gallbladder cancer is a relatively uncommon human malignant tumor with an extremely poor prognosis. Currently, no biomarkers can accurately diagnose gallbladder cancer and predict patients’ prognosis. XRCC1 is involved in tumorigenesis, progression, and chemo-resistance of several human cancers, but the role of XRCC1 in gallbladder cancer is never reported. In this study, we investigated the expression of XRCC1 and its clinicopathological and prognostic significance in gallbladder cancer, and explored the biological role of XRCC1 in gallbladder cancer cells. We found that XRCC1 was significantly up-regulated in gallbladder cancer in protein and mRNA levels. Positive XRCC1 expression was correlated with aggressive clinicopathological features and was an independent poor prognostic factor in gallbladder cancer. The ROC curves suggested that XRCC1 expression had potential clinicopathological diagnostic value in gallbladder cancer. *In vitro*, XRCC1 was overexpression in CD133^+^GBC-SD cells compared to GBC-SD cells. In functional experiment, XRCC1 knockdown had a non-significant impact on proliferation, migration, invasion, and apoptosis of CD133^+^GBC-SD cells. But, XRCC1 knockdown could significantly improve the sensitivity of CD133^+^GBC-SD cells to 5-Fluorouracil via promoting cell necrosis and apoptosis. Thus, this study indicates that XRCC1 may be a promising predictive biomarker of gallbladder cancer and a potential therapeutic target for gallbladder cancer.

## Introduction

Gallbladder cancer (GBC) is a relatively uncommon human malignant tumor with an extremely poor prognosis. Histologically, GBC mainly consists of gallbladder adenocarcinoma (AC) (about 90%) and squamous cell/adenosquamous carcinoma (SC/ASC) (accounting for 1–12%) (Roa et al., 2011; Samuel et al., 2018). Although various studies about GBC were performed, GBC clinical outcome remains extremely poor. Currently, radical resection remains the only way to cure GBC, although adjuvant treatments (chemotherapy and radiotherapy) are available. Because GBC patients at early stages present asymptomatic and are difficult to be diagnosed, most patients are diagnosed at late stages when patients lost the chance to receive radical surgery (Reid et al., 2007; Henley et al., 2015). Additionally, GBC is often resistant to chemotherapy and radiotherapy (Horgan et al., 2012). These above reasons make the prognosis of GBC unsatisfactory. Although many biological marks have been studied, no one can accurately diagnose GBC and predict patients’ survival (Sicklick et al., 2016; Sharma et al., 2017). Therefore, discovering reliable early diagnostic biomarkers and exploring the mechanism of treatment resistance are critically important to improve the prognosis of GBC.

DNA repair pathways are related to tumorigenesis and treatment resistance, and base excision repair (BER) is one pathway of DNA repair systems (Wood et al., 2001). BER functions an essential role in protecting the genome against chemical carcinogens and ionizing radiations (Tudek, 2007). Chemotherapy and radiotherapy usually kill tumor cells by causing DNA damage which could be repaired via BER. As a BER protein, x-ray repair cross-complementing group 1 (XRCC1), a 70-kDa protein, is encoded by the gene located on chromosome 19q13.2–13.3 (Thompson and West, 2000). XRCC1 functions as a scaffold protein in the BER and its aberrant expression is associated with carcinogenesis of multiply human malignant tumors (Hanssen-Bauer et al., 2012; Meng et al., 2017; Mei et al., 2019). Currently, researches on XRCC1 mainly concentrate on the relationship between gene polymorphism and cancer susceptibility. Recently, several studies have investigated the clinicopathological and prognostic significance of XRCC1 in human cancers including gastric cancer, ovarian cancer, and non-small cell lung cancer (Wang et al., 2012; Abdel-Fatah et al., 2013; Liu et al., 2015). Moreover, XRCC1 affects the effectiveness of chemotherapy and the role of XRCC1 in chemosensitivity varies in different types of cancer. For example, in gastric and ovarian cancer, patients with low XRCC1 expression exhibited favorable response to platinum-based chemotherapy (Wang et al., 2012; Abdel-Fatah et al., 2013). However, bladder cancer patients with high XRCC1 expression had a favorable chemosensitivity to platinum-based chemotherapy (Sakano et al., 2013). As we know, the role of XRCC1 in GBC is never reported.

Therefore, in this study, we investigated the expression of XRCC1 and its clinicopathological and prognostic significance in gallbladder SC/ASC and AC. Furthermore, the biological function of XRCC1 in CD133^+^GBC cells was evaluated.

## Materials and Methods

### Case Selection

This study was approved by the Ethics Committee for Human Research, Central South University and performed in accordance with the Declaration of Helsinki. The included patients were histologically diagnosed by two pathologists. These patients never received chemotherapy or radiotherapy preoperatively and postoperatively. We collected 69 SC/ASC samples from January 2001 to December 2013 (16 from Xiangya Hospital, 31 from Second Xiangya Hospital, 10 from Third Xiangya Hospital, 5 from Hunan Provincial People Hospital, 5 from Hunan Provincial Tumor Hospital, and 1 each from Changde Central Hospital and Loudi Central Hospital). According to the recommendations of the American Joint Committee on Cancer, tumors with a squamous component ≥10% were considered as ASC. The 69 SC/ASCs accounted for 5.5% of 1248 GBCs. We collected 146 AC samples from January 2008 and December 2013 at Second Xiangya Hospital and Third Xiangya Hospital. Survival data for these patients was obtained through letters and telephone calls. The follow-up time was 2 years, and patients who survived over 2 years were considered as censored cases.

### EnVision Immunohistochemistry

The rabbit anti-human XRCC1 primary antibody and HRP-conjugated anti-rabbit second antibody were purchased from Santa Cruz Biotechnology (CA, United States). EnVisionTM Detection Kit was purchased from Dako Laboratories (CA, United States). Immunohistochemistry was performed as previously described (Wu et al., 2017). Briefly, four-micrometer-thick sections were cut from routinely paraffin-embedded tissues. The sections were deparaffinized and then incubated with peroxidase inhibitor (3% H_2_O_2_) in the dark for 15 min, followed by EDTA-trypsin digestion for 15 min. Then, the sections were incubated with primary antibody for 60 min at 37°C. Next, the sections were incubated with the second antibody for 30 min at 37°C after being soaked with PBS for 3 × 5 min. Then, solution A was added to the sections for 30 min, followed by DAB staining and hematoxylin counter-staining. The slides were dehydrated with different concentrations (70%–100%) of alcohol, and soaked in xylene for 3 × 5 min and finally mounted with neutral balsam.

### Evaluation of Immunostaining

Ten random fields were examined per section by two independent pathologists. The percent of positively stained cells was determined. Strength of staining was rated on a scale of 1 to 3 (1: little to no positive staining or uncertainly weak staining; 2: weak to moderate staining; 3: moderate to strong staining). A section was determined as positive expression when the percent of positively stained cells was ≥10% and staining strength was ≥2. The few sections where percent positive staining was 5% to 10% and staining strength was 3 were also regarded as positive.

### Western Blot

Total protein was extracted from frozen tissues or cell samples. Protein concentrations were tested via a BCA protein-assay. Protein samples were separated on 10% SDS-PAGE gel. The separated proteins were transferred to Immun-Blot PVDF membrane (Bio-Rad) using a wet transfer system (Bio-Rad). The membrane was blocked with 5% skimmed milk and then incubated with primary antibody (XRCC1, 1:500, proteintech, China) at 4°C overnight, followed by incubation with HRP-linked anti-rabbit IgG (Merck Millipore) in a dilution of 1: 10000 for 1 h at room temperature.

### Real-Time Quantitative PCR (qRT-PCR)

Trizol reagent (Beijing Dingguo Changsheng Biotech, Co., Ltd., China) was applied to extract total RNA. The RNA was reverse-transcribed to cDNA by the PrimeScript RT reagent Kit (Takara Biomedical Tech, Co., Ltd., China). The cDNA was subjected to qRT-PCR using SYBR Premix Ex Taq II (Takara, Co., Ltd., China) and the assay was performed on the CFX connect system (Bio-Rad Co., Ltd., United States). GAPDH was used as an internal control. The primers were synthesized from Tsingke Biological Technology Co., (Changsha, Hunan, China), and sequences of primers were listed as followed: XRCC1: Forward 5′-CCTTTGGCTTGAGTTTTGTACG-3′, Reverse 5′-CCTCCTTCACACGGAACTGG-3′; GAPDH: Forward 5′-ATGACCACAGTCCATGCCATCA-3′, Reverse 5′-TTACTCCTTGGAGGCCATGTAG-3′.

### Cell Lines and Culture

The human gallbladder cancer cell line GBC-SD was obtained from the Cell Bank of the Chinese Academy of Sciences (Shanghai, China). Cells were cultured in RPMI-1640 (Hyclone, United States) supplemented with 10% fetal bovine serum (Gibco, Grand Island, NY, United States), Penicillin 100 U/ml and Streptomycin 100 ug/ml (Beyotime, China) in humidified atmosphere at 37°C and 5% CO_2_.

### Isolation of CD133^+^cell Population by Magnetic Cell Sorting

For magnetic cell sorting, cells were labeled with CD133 microbeads and sorted using the Miltenyi Biotec CD133 Cell Isolation Kit according to the manufacturer’s protocols (Miltenyi Biotec, Germany). Magnetic separation was performed twice to obtain high purity of CD133^+^cells. The purity of sorted cells was evaluated by flow cytometry with a FACS Calibur machine after labeling with phycoerythrin (PE)-conjugated anti-human CD133 antibody (Biolegend, United States).

### Inhibition of XRCC1 Expression by shRNA Transfection

XRCC1 shRNA and negative control shRNA were purchased from GeneChem (Shanghai, China). XRCC1 shRNA or negative control shRNA were mixed with RPIM-1640 (Hyclone, United States) and Lip2000 (Invitrogen, United States), and then incubated at room temperature for 20 min. Approximately 2 × 10^5^ CD133^+^cells were plated in 6-well plates, followed by treating them with the transfection mixture and incubated at 37°C with 5% CO_2_. Cells were harvested at 6 h post-transfection for further studies.

### CCK8 Assays

The proliferation of CD133^+^cells transfected with control shRNA or XRCC1 shRNA was detected by use of Cell Counting Kit-8 (CCK8) (DOJINDO, Japan). Cells were seeded into 96-well culture plates at a density of 1 × 10^4^ cells/100 ul. Four wells of each group were detected every day. At the end of each experiment, CCK-8 solution was added to each well, and the cultures were incubated at 37°C for 4 h. Then, the cultures were detected by use of a microplate reader.

### Transwell Assays

Cell migration assays were performed in a 24-well Transwell plate (Corning, United States). Cells in serum-free medium (1 × 10^5^ cells) was added to the upper chamber. Complete medium was added to the bottom wells of the chamber. After 48 h of incubation at 37°C, the cells that did not migrate were removed from the upper face of the filters. The number of cells migrating to the lower face was counted after fixed with 4% formaldehyde and stained with 0.5% crystal violet. The number of cells was counted under a microscope. The cell invasion assay was essentially the same as the migration assays, except that the membrane filters were coated with Matrigel (Becton, Dickinson and Company, United States).

### Flow Cytometry Assay for Apoptosis

Cells transfected with control shRNA or XRCC1 shRNA were cultured in a 6-well plate. After 48 h, cells were harvested by trypsinization, washed twice with PBS, and then stained with annexin V-APC (APC) (NanJing KeyGen Biotech, Co., Ltd., China) and propidium iodide (PI) (NanJing KeyGen Biotech, Co., Ltd., China) to detect cell apoptosis. Samples were immediately detected in the flow cytometer. This method can distinguish the cells in early (APC+/PI-) and late (APC+/PI+) apoptosis.

### Drug Sensitivity Assay

CD133^+^cells transfected with XRCC1 shRNA or negative control shRNA were cultured in 96 well plates (1 × 10^4^ cells/well) overnight. On the second day, the cells were treated with 5-Fluorouracil (5-FU, final concentration of 0.1 mg/L) (APExBIO, United States) (Paschall et al., 2016). After 72 h, CCK-8 solution was added to each well, and the cultures were incubated at 37°C for 4 h. Then, the cultures were detected by use of a microplate reader.

CD133^+^cells transfected with XRCC1 shRNA or negative control shRNA were cultured in 6 well plates (2 × 10^5^ cells/well) overnight. On the second day, the cells were treated with 5-Fluorouracil (5-FU, final concentration of 0.1 mg/L). After 72 h, cells were harvested to assess cell apoptosis by flow cytometry as above.

### Statistical Analysis

Data was analyzed using SPSS 13.0. The relationship between XRCC1 expression and clinicopathological factors was analyzed using χ^2^ or Fisher’s exact test. Kaplan-Meier and Log-rank test were used for univariate survival analysis. Cox proportional hazards model was used for univariate and multivariate analysis. A *P* < 0.05 was considered as statistical significance.

## Results

### Characteristics of Patients

Among the 69 SC/ASC samples, 44 were collected from female patients and patient ages ranged from 35 to 80 (53.8 ± 10.2) years. Among the 146 AC patients, 85 were female with an age range of 33 to 78 (52.4 ± 9.6) years. The detail clinicopathological information of the 146 SC/ASC patients and the 69 AC patients was presented in Table 1. Briefly, among the 69 SC/ASCs, the squamous cell component presented well-differentiated in 19 (27.5%), moderately differentiated in 33 (47.8%), and poorly differentiated in 17 (24.6%). The 146 ACs consisted of 51 well-differentiated types (34.9%), 54 moderately differentiated types (37.0%) and 41 poorly differentiated types (28.1%). Among the SC/ASC patients, invasion to surrounding tissues and organs was observed in 45 patients (65.2%); 42 (60.7%) occurred regional lymph node metastasis; and 38 (55.1%) existed gallstones. Among the 146 AC patients, 74 (50.7%) occurred invasion; 66 (45.2%) presented regional lymph node metastasis; and 68 (46.6%) had gallstones. According to tumor-node-metastasis (TNM) staging, 29 SC/ASCs and 40 SC/ASCs stage I + II and stage III + IV, respectively. Among the 146 ACs, 77 were in a stage of I or II and 69 were in a stage of III or IV. Among all patients, 27 SC/ASC patients and 75 AC patients received radical surgery; 28 SC/ASC patients and 50 AC patients received palliative surgery; 14 SC/ASC patients and 21 AC patients only underwent biops.

**TABLE 1:** Comparison of gallbladder SC/ASC and AC clinicopathological characteristics and XRCC1 expression status.

**Clinicopathological characteristics**	**Number of SC/ASC (%)**	**Number of AC (%)**	***P***
Gender			
Male	25 (36.2)	61 (41.8)	0.438
Female	44 (63.8)	85 (58.2)	
Age			
≤45 years	3 (4.3)	20 (13.7)	0.038
>45 years	66 (95.7)	126 (86.3)	
Differentiation			
Well	19 (27.5)	51 (34.9)	0.308
Moderate	33 (47.8)	54 (37.0)	
Poor	17 (24.6)	41 (28.1)	
Maximum tumor diameter			
≤3 cm	39 (56.5)	90 (61.6)	0.474
>3 cm	30 (43.5)	56 (38.4)	
Cholecystolithiasis			
No	31 (44.9)	78 (53.4)	0.245
Yes	38 (55.1)	68 (46.6)	
TNM stages			
I + II	29 (42.0)	77 (52.7)	0.143
III + IV	40 (58.0)	69 (47.3)	
Lymph node metastasis			
No	27 (39.1)	80 (54.8)	0.032
Yes	42 (60.9)	66 (45.2)	
Locoregional invasion			
No	24 (34.8)	72 (49.3)	0.045
Yes	45 (65.2)	74 (50.7)	
Surgical methods			
Radical	27(39.1)	75 (51.4)	0.223
Palliative	28 (40.6)	50 (34.2)	
Without resection	14 (20.3)	21 (14.4)	
XRCC1			
−	28 (40.6)	58 (39.7)	0.905
+	41 (59.4)	88 (60.3)	

*−, negative expression; +, positive expression.*

### XRCC1 Is Significantly Over-Expressed in Gallbladder Cancer Tissues

**FIGURE 1:**
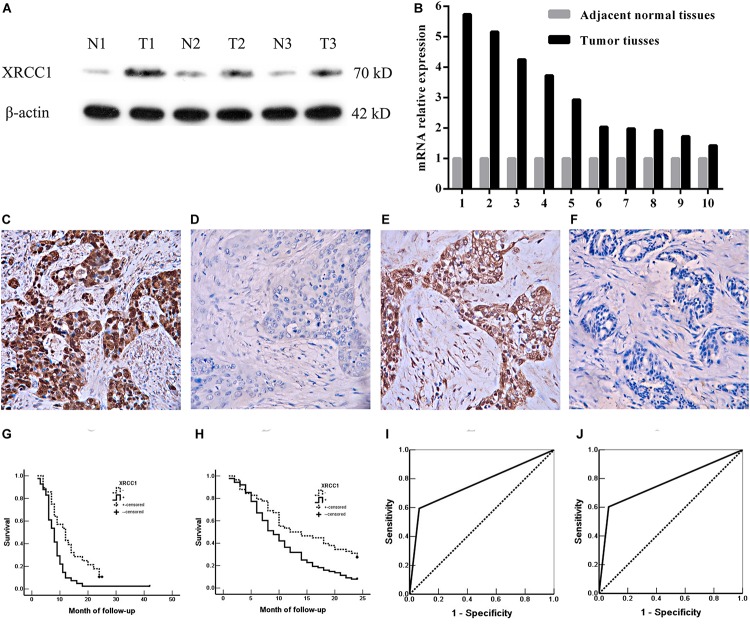
XRCC1 expression is up-regulated in GBC and associated with poor prognosis. (**A**) XRCC1 expression was analyzed by western blot. (**B**) XRCC1 expression was assessed by qRT-PCR. (**C**) Positive expression of XRCC1 in moderately differentiated ASC, ×200. (**D**) Negative expression of XRCC1 in moderately differentiated SC, ×200. (**E**) Positive expression of XRCC1 in moderately differentiated AC, ×200. (**F**) Negative expression of XRCC1 in well differentiated AC, ×200. (**G**) Kaplan-Meier curves for SC/ASC patients with positive and negative XRCC1 expression. (**H**) Kaplan-Meier curves for AC patients with positive and negative XRCC1 expression. (**I**) ROC of diagonal segments was produced by ties of XRCC1 in SC/ASC. (**J**) ROC of diagonal segments was produced by ties of XRCC1 in AC.

To evaluate the expression of XRCC1 in GBC tissues and corresponding adjacent non-tumor tissues, qRT-PCR and western blot were performed. The results demonstrated that XRCC1 expression in GBC tissues was significantly higher than adjacent non-tumor tissues both in mRNA and protein levels (Figures 1A,B).

We then assessed XRCC1 expression in gallbladder cancer tissues (including 69 SC/ASCs and 146 ACs) and gallbladder epithelium with chronic cholecystitis by immunohistochemistry. The majority of XRCC1 positive-reaction was localized in the cytoplasm of the SC/ASC (Figure 1C) and AC (Figure 1E). The representative images of XRCC1 negative expression in SC/ASC and AC were seen in Figure 1D and Figure 1F, respectively. The staining positive rate was significantly higher in SC/ASC (59.4%) and AC (60.3%) than gallbladder epithelium with chronic cholecystitis (6.7%, *P* < 0.01). The epithelium of chronic cholecystitis with high XRCC1 expression showed moderate to severe dysplasia. This suggested that XRCC1 may be a biomarker to evaluate the pre-malignant changes.

### Comparison of Gallbladder ASC/SC and AC in Clinicopathological Features Including XRCC1 Expression

As showed in Table 1, the percentage of cases with a patient age over 45 years, lymph node metastasis and invasion was significantly higher in SC/ASC compared with AC (all *P* < 0.05). However, there was a non-significant difference between SC/ASC and AC in other clinicopathological features including tumor differentiated degree, tumor size, TNM stages, receiving surgical methods, and XRCC1 positive expression (all *P* > 0.05, Table1).

### XRCC1 Positive Expression Correlates With Poor Clinicopathological Features of Gallbladder SC/ASC and AC Patients

We further evaluated the clinicopathological significance of XRCC1 expression in SC/ASC and AC patients. We found that XRCC1 positive expression was associated with several poor clinicopathological features of gallbladder cancer. In SC/ASC, XRCC1 positive expression was positively correlated with lymph node metastasis, invasion, and only receiving biopsy (all *P* < 0.05, Table 2). Similarly, XRCC1 positive expression was positively associated with large tumor size (>3 cm), lymph node metastasis, invasion, late TNM stages (III + IV), only receiving biopsy in AC (all *P* < 0.05, Table 2).

**TABLE 2:** Correlations of XRCC1 expression with the clinicopathological characteristics of gallbladder SC/ASC and AC.

**Clinicopathological characteristics**	**SC/ASC**			**AC**		
	**Number of patients**	**Positive Number (%)**	***P***	**Number of patients**	**Positive Number (%)**	***P***
Differentiation						
Well	19	10 (52.6)	0.738	51	29 (56.9)	0.131
Moderately	33	21 (63.6)		54	29 (53.7)	
Poorly	17	10 (58.8)		41	30 (73.2)	
Tumor size						
≤3cm	39	16 (53.3)	0.366	90	45 (50.0)	0.001
>3cm	30	25 (64.1)		56	43 (76.8)	
Gallstone						
No	31	18 (58.1)	0.836	78	52 (66.7)	0.091
Yes	38	23 (60.5)		68	36 (52.9)	
Lymph node metastasis						
No	27	12 (44.4)	0.037	80	40 (50.0)	0.005
Yes	42	29 (69.1)		66	48 (72.7)	
Invasion						
No	24	10 (41.7)	0.028	72	37 (51.4)	0.030
Yes	45	31 (68.9)		74	51 (68.9)	
TNM stage						
I + II	29	14 (48.3)	0.108	77	38 (49.4)	0.004
III + IV	40	27 (67.5)		69	50 (72.5)	
Surgery						
Radical	27	11 (40.7)	0.031	75	39 (52.0)	0.006
Palliative	28	19 (67.9)		50	30 (60.0)	
Biopsy	14	11 (78.6)		21	19 (90.5)	

### XRCC1 Positive Expression Is an Independent Risk Factor for the Prognosis of Gallbladder SC/ASC and AC Patients

Gallbladder cancer patients (both AC/ASC and AC) in XRCC1 positive expression group had significantly shorter average survival time than patients in the negative expression group (all *P* < 0.01, Table 3). The Kaplan-Meier survival curves demonstrated that patients with XRCC1 positive expression had a poor overall survival than patients with XRCC1 negative expression (Figures 1G,H). Moreover, univariate and multivariate analysis showed that XRCC1 positive expression was an independent risk factor for the overall survival of gallbladder SC/ASC and AC patients (Tables 4, 5). Finally, the receiver operating characteristic (ROC) curve was depicted to assess the diagnostic efficacy of XRCC1 expression in SC/ASC and AC. The AUC of XRCC1 expression in SC/ASC and AC was 0.764 (95%CI: 0.669–0.859) and 0.768 (95%CI: 0.689–0.847) respectively (Figures 1I,J). These results fully revealed that XRCC1 was closely related to poor survival and might be a novel independent prognosis biomarker for gallbladder SC/ASC and AC patients.

**TABLE 3:** Relationship between XRCC1 expression, clinicopathological characteristics and average survival of SC/ASC and AC patients.

**Clinicopathological characteristics**	**SC/ASC**			**AC**		
	**Sample (n)**	**Average survival (month)**	***P***	**Sample (n)**	**Average survival (month)**	***P***
Differentiation						
Well	19	13.68(5−24)	0.000	51	16.69(5−24)	0.000
Moderately	33	11.58(4−24)		54	12.33(2−24)	
Poorly	17	6.12(2−14)		41	6.49(1−24)	
Tumor size						
≤3cm	30	14.57(6−24)	0.000	90	14.60(1−24)	0.000
>3cm	39	7.44(2−24)		56	8.38(1−24)	
Gallstones						
No	31	8.26(3−18)	0.008	78	12.19(2−24)	0.980
Yes	38	12.90(2−24)		68	12.24(1−24)	
TNM stage						
I + II	29	16.31(3−24)	0.000	77	16.99(3−24)	0.000
III + IV	40	6.83(2−14)		69	6.88(1−24)	
Lymph node metastasis						
No	27	16.04(3−24)	0.000	80	16.35(2−24)	0.000
Yes	42	7.45(2−15)		66	7.20(1−24)	
Invasion						
No	24	17.25(3−24)	0.000	72	18.08(4−24)	0.000
Yes	45	7.38(2−20)		74	6.50(1−14)	
Surgery						
Radical	27	16.93(5−24)	0.000	75	17.84(6−24)	0.000
Palliative	28	7.32(2−12)		50	6.86(1−14)	
Biopsy	14	6.00(4−8)		21	4.86(1−9)	
XRCC1						
−	28	12.95(4−24)	0.002	58	14.47(2−24)	0.001
+	41	8.42(2−24)		88	10.73(1−24)	

*−, negative expression; +, positive expression.*

**TABLE 4:** Univariate Cox regression analysis of survival rate in SC/ASC and AC patients.

**Groups**	**Factors**	**SC/ASC**		**AC**	
		***P***	**HR (95% CI)**	***P***	**HR (95% CI)**
Differentiated degree	Well/moderately/poorly	0.000	2.040(1.394−2.983)	0.000	2.227(1.740−2.851)
Tumor size	≤3 cm/>3 cm	0.034	1.765(1.044−2.984)	0.000	2.331(1.614−3.367)
Gallstone	No/Yes	0.088	1.565(0.935−2.261)	0.981	1.004(0.704−1.433)
TNM stage	I + II/III + IV	0.000	6.830(3.619−12.890)	0.000	5.923(3.898−9.002)
Lymph node metastasis	No/Yes	0.000	4.550(2.453−8.438)	0.000	5.021(3.312−7.612)
Invasion	No/Yes	0.000	5.453(2.942−10.104)	0.000	12.808(7.412−22.131)
Surgery	Radical/Palliative/Biopsy	0.000	4.240(2.709−6.637)	0.000	5.693(4.081−7.940)
XRCC1	−/+	0.005	2.125(1.258−3.591)	0.002	1.826(1.251−2.666)

*Abbreviation: HR, hazard risk ratio; CI, confidence interval; −, negative expression; +, positive expression.*

**TABLE 5:** Multivariate Cox regression analysis of survival rate in SC/ASC and AC patients.

**Groups**	**Factors**	**SC/ASC**		**AC**	
		***P***	**HR (95% CI)**	***P***	**HR (95% CI)**
Differentiated degree	Well/moderately/poorly	0.005	1.815(1.198−2.750)	0.002	1.514(1.158−1.981)
Tumor size	≤3 cm/>3 cm	0.030	1.974(1.067−3.653)	0.016	1.772(1.111−2.825)
Gallstone	No/Yes	0.461	1.237(0.702−2.180)	0.460	1.153(0.791−1.679)
TNM stage	I + II/III + IV	0.024	3.662(1.189−11.280)	0.002	2.965(1.499−5.865)
Lymph node metastasis	No/Yes	0.002	3.823(1.607−9.091)	0.000	3.869(2.062−7.258)
Invasion	No/Yes	0.016	3.684(1.273−10.658)	0.000	6.488(3.287−12.809)
Surgery	Radical/Palliative/Biopsy	0.016	1.960(1.132−3.393)	0.000	2.284(1.522−3.427)
XRCC1	−/+	0.020	1.998(1.116−3.576)	0.011	1.721(1.134−2.613)

*HR, hazard risk ratio; CI, confidence interval; −, negative expression; +, positive expression.*

### XRCC1 Is Significantly Up-Regulated in CD133^+^GBC-SD Cells Compared With Normal GBC-SD Cells

**FIGURE 2:**
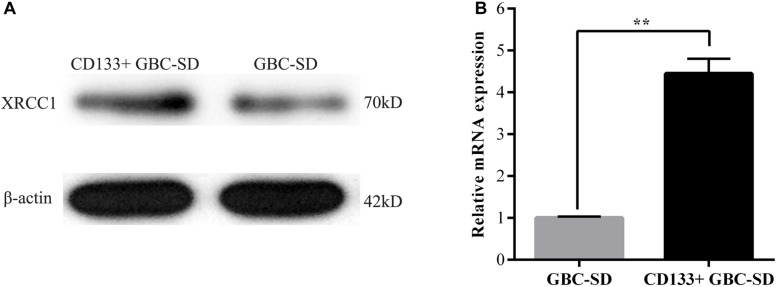
XRCC1 is up-regulated in CD133^+^ GBC-SD cells. (**A**) XRCC1 expression in GBC-SD cells and CD133^+^GBC-SD cells was analyzed by western blotting. (**B**) XRCC1 expression in GBC-SD cells and CD133^+^GBC-SD cells was assessed by qRT-PCR (**P < 0.01).

Previous studies reported that both XRCC1 and CD133^+^cancer cells are related to tumor drug resistance so that we studied the role of XRCC1 in CD133^+^GBC-SD cells. CD133^+^GBC-SD cells were obtained from GBC-SD cells by CD133 magnetic bead sorting. We applied qRT-PCR and western blot to research XRCC1 expression in normal GBC-SD cells and CD133^+^GBC-SD cells. Compared with GBC-SD cells, XRCC1 mRNA and protein were overexpressed in CD133^+^GBC-SD cells (Figure 2). Based on previous studies, these results indicated that XRCC1 might affect the unique biological features of CD133^+^GBC-SD cells compared to normal GBC-SD cells, such as chemo-resistance.

### Knockdown XRCC1 Has a Non-significant Effect on CD133^+^GBC-SD Cells Proliferation, Migration, Invasion, and Apoptosis

**FIGURE 3:**
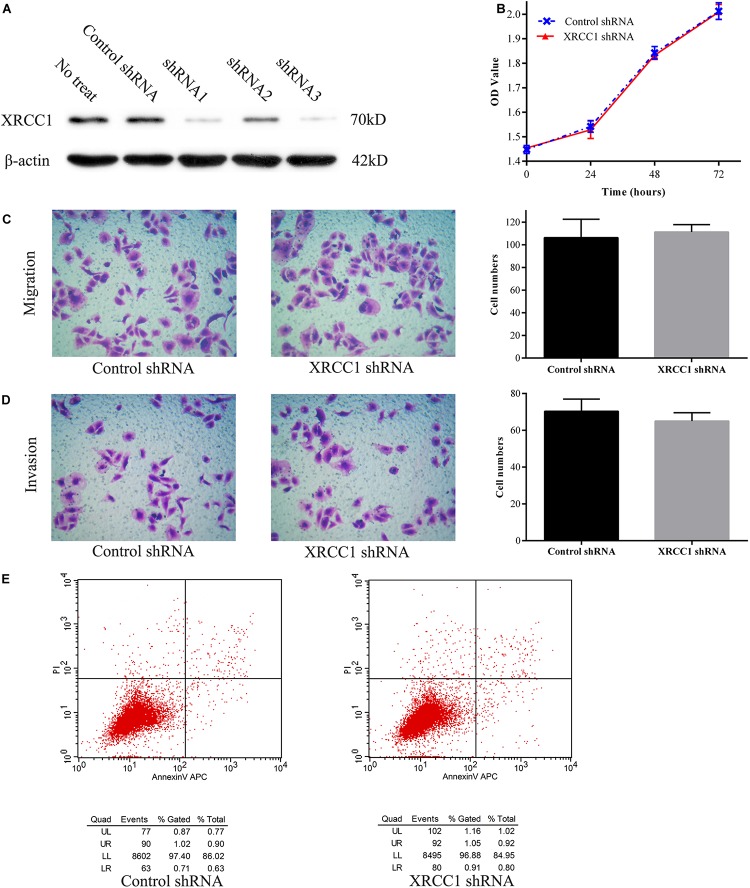
XRCC1 has no effect on proliferation, migration, invasion, and apoptosis of CD133^+^GBC-SD cells. (**A**) Western blot detected XRCC1 expression in CD133^+^GBC-SD cells transfected with different shRNAs. (**B**) Proliferation of XRCC1-shRNA/CD133^+^GBC-SD cells and control-shRNA/CD133^+^GBC-SD cells was examined by CCK8. (**C**) Transwell migration assay detected the migration capacity of XRCC1-shRNA/CD133^+^GBC-SD cells and control-shRNA/CD133^+^GBC-SD cells. (**D**) Transwell invasion assay detected the invasion capacity of XRCC1-shRNA/CD133^+^GBC-SD cells and control-shRNA/CD133^+^GBC-SD cells. (**E**) Flow cytometer detected the apoptosis capacity of XRCC1-shRNA/CD133^+^GBC-SD cells and control-shRNA/CD133^+^GBC-SD cells; UL: necrosis, UR: late apoptosis, LL: normal cells, LR: early apoptosis.

To further study the function of XRCC1 in CD133^+^GBC-SD cells, XRCC1 expression in cells was manipulated via short hairpin RNA (shRNA) knockdown. Three shRNAs (shRNA1, shRNA2, and shRNA3) were designed to knockdown XRCC1 expression in CD133^+^GBC-SD cells. After CD133^+^GBC-SD cells were infected with XRCC1-shRNA, the expression level of XRCC1 was tested by western blotting to evaluate the efficacy of shRNA knockdown. Among the three XRCC1-shRNAs, shRNA3 was the most effective one (Figure 3A) and was selected for further studies. To study the effect of XRCC1 knockdown on the proliferation, migration, invasion, and apoptosis of CD133^+^GBC-SD cells, CCK8 assay, transwell assay, and flow cytometry were performed. Our results showed that XRCC1 knockdown in CD133^+^GBC-SD cells had a non-significant impact on the ability of proliferation, migration, invasion, and apoptosis, compared with control-shRNA/CD133^+^GBC-SD cells (Figure 3).

### XRCC1 Facilitates CD133^+^GBC-SD Cells Resistance to 5-FU

**FIGURE 4:**
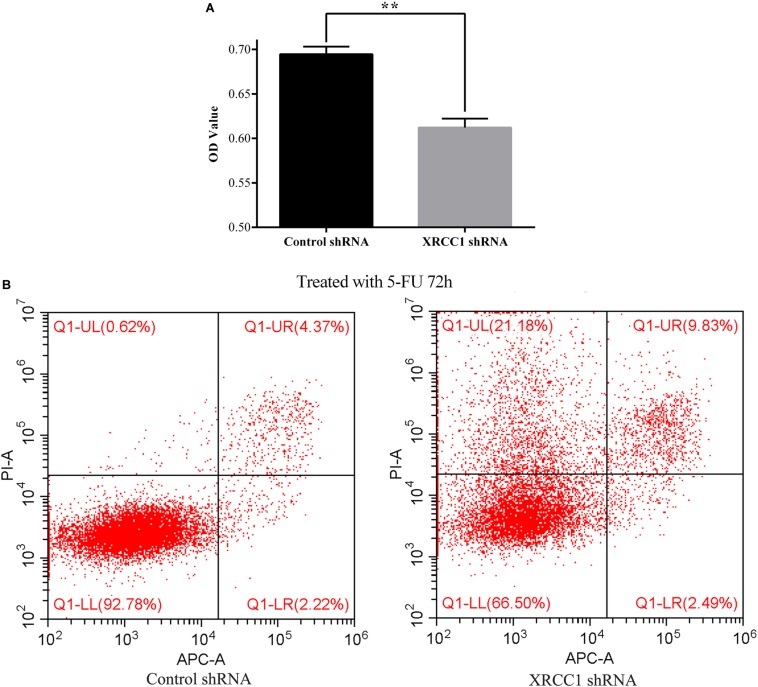
XRCC1 is related to CD133^+^GBC-SD cells resistance to 5-FU. (**A**) Cell viability of cells treated with 5-FU 72 h was assessed by CCK8 (**P < 0.01). (**B**) Apoptosis of cells treated with 5-FU 72 h was examined by Flow cytometer; Q1-UL: necrosis cells, Q1-UR: late apoptosis cells, Q1-LL: normal cells, Q1-LR: early apoptosis cells.

To explore the role of XRCC1 in CD133^+^GBC-SD cells drug resistance, 5-FU was used to treat control-shRNA/CD133^+^GBC-SD cells and XRCC1-shRNA/CD133^+^GBC-SD cells. After treated with 5-FU (0.1 mg/L) for 72 h, CCK8 assay was performed to assess the cell totality of every group. Results showed that in CCK8 assay, XRCC1-shRNA/CD133^+^GBC-SD had a lower absorbance compared to control-shRNA/CD133^+^GBC-SD, which suggested that XRCC1 could promote CD133^+^GBC-SD cell resistance to 5-FU (Figure 4A). To further validate the results of CCK8 assay, flow cytometry was performed. Under 5-FU treated 72 h, flow cytometry revealed that cell necrosis and apoptosis were significantly increased in XRCC1-shRNA/CD133^+^GBC-SD compared to control-shRNA/CD133^+^GBC-SD (Figure 4B). Thus, our results indicated that XRCC1 might promote CD133^+^GBC-SD cells resistance to 5-FU through inhibiting cell necrosis and apoptosis.

## Discussion

GBC is an aggressive malignant of the biliary tree and consists of several pathological subtypes including AC and SC/ASC. In comparison of AC, the incidence rate of gallbladder SC/ASC is relatively rare and its clinicopathological features remain to be further elucidated. Currently, most reports investigated SC/ASC based on individual cases or small case samples. As far as we know, the 69 SC/ASC cases that we included in this study are relatively large samples in current clinical studies on gallbladder SC/ASC, which could provide more detail clinicopathological knowledge about SC/ASC. In the present study, we found that SC/ASC accounted for 5.5% of GBC and the occurring rate of lymph node metastasis and invasion was significantly higher in SC/ASC than AC, which was consistent with previous reports (Kim et al., 2011; Roa et al., 2011; Samuel et al., 2018). In agreement with previous researches (Chan et al., 2007; Kim et al., 2011), our results also showed that gallbladder SC/ASC and AC had similar clinicopathological features such as tumor differentiated degree, tumor size, the existence of gallstone, TNM stage, and XRCC1 expression.

Nowadays, the prognosis of GBCs remains extremely poor. In this study, our data revealed that lymph node metastasis, invasion, large tumor size, and advanced TNM stages were independent risk factors for patient’s survival, and radical surgery could significantly prolong the mean survival time of patients in SC/ASC and AC. These results suggested that early diagnosis was very important for improving the clinical prognosis of GBC. Thus, it is extremely vital to discover early specific diagnostic biomarkers and explore the reason why GBC resists to chemotherapy. Previous works have demonstrated that XRCC1 is associated with tumor resistance to chemotherapy and radiotherapy, carcinogenesis, and tumor progression (Sak et al., 2005; Hanssen-Bauer et al., 2012; Xu et al., 2014; Li et al., 2018). CD133^+^cancer cells are a small subgroup of tumor cells and related to tumor resistance to chemotherapy and radiotherapy (Zhang et al., 2010; Desai et al., 2014; Vincent et al., 2014; Kanwal et al., 2018). Thus, we further studied the clinicopathological and prognostic significance of XRCC1 in gallbladder SC/ASC and AC, and evaluated the biological role of XRCC1 in CD133^+^ GBC-SD cells.

As a DNA repair gene, XRCC1 is involved in tumorigenesis, progression, and poor prognosis of many human cancer types. In this study, we observed that XRCC1 expression was up-regulated in GBC compared with non-tumor tissues, which was consistent with previous studies where XRCC1 was overexpression in ovarian cancer and head and neck squamous cell cancer (Ang et al., 2011; Abdel-Fatah et al., 2013). On the contrary, several reports showed that XRCC1 was down-regulated in glioma, bladder cancer, pancreatic cancer, and gastric cancer (Crnogorac-Jurcevic et al., 2002; Sak et al., 2005; Wang et al., 2012; Mei et al., 2019). This contradiction may be owed to the organ specificity. Furthermore, we found that the epithelium of chronic cholecystitis with high XRCC1 expression showed moderate to severe dysplasia, suggesting that XRCC1 may be involved in the processes that benign lesions evolve into GBC. Thus, we further evaluated the clinicopathological and prognostic significance of XRCC1 in gallbladder SC/ASC and AC. Our data demonstrated that XRCC1 positive expression was significantly related to lymph node metastasis, invasion, and poor prognosis, which was consistent with previous studies (Ang et al., 2011; Abdel-Fatah et al., 2013; Mian et al., 2016). Moreover, cox univariate and multivariate analysis further showed that XRCC1 positive expression was an independent risk factor for the overall survival of SC/ASC and AC. The AUC of XRCC1 indicated that the expression of XRCC1 might have potential clinicopathological diagnostic significance in SC/ASC and AC. These results suggested that XRCC1 might be involved in carcinogenesis and development of GBC.

Previous studies have demonstrated that XRCC1 plays a role in regulating cell biological features such as proliferation, migration, invasion, and drug resistance in several human cancer cell lines (Xu et al., 2014; Meng et al., 2017; Li et al., 2018; Mei et al., 2019). However, there is no study reporting the function of XRCC1 in gallbladder cancer cells. Herein, we firstly studied the biological role of XRCC1 in CD133^+^GBC-SD cells. Unexpectedly, the functional experiments revealed that knockdown of XRCC1 had no significant effect on the ability of proliferation, migration, invasion, and apoptosis in CD133^+^GBC-SD cells, which was inconsistent with previous researches (Li et al., 2018; Mei et al., 2019). This inconsistency may be caused by cell specificity. Additionally, we found that XRCC1 was up-regulated in CD133^+^GBC-SD cells compared with GBC-SD cells, indicating that XRCC1 might be associated with unique biological features of CD133^+^cancer cells, such as chemo-resistance. Therefore, we further investigated the impact of XRCC1 on CD133^+^GBC-SD cells resistance to 5-FU. As we suspected, our results showed that XRCC1 were contributed to the resistance of CD133^+^GBC-SD cells to 5-FU via inhibiting cell necrosis and apoptosis, which was in accordance with previous studies (Abdel-Fatah et al., 2013; Xu et al., 2014). Thus, XRCC1 may promote GBC resistance to chemotherapy, which needs further studies to validate and explore potential molecular mechanism. Thus, we speculated that XRCC1 might be a promising target to improve the sensitivity of GBC to chemotherapy.

## Conclusion

In conclusion, this study demonstrated that XRCC1 was overexpression in gallbladder cancer tissues. XRCC1 positive expression was associated with aggressive clinicopathological features and poor prognosis of gallbladder SC/ASC and AC. Moreover, XRCC1 was related to the chemo-resistance of CD133^+^GBC-SD cells to 5-FU. Thus, XRCC1 may be a promising predictive biomarker and a potential therapeutic target for GBC.

## Data Availability Statement

The datasets generated for this study are available on request to the corresponding author.

## Ethics Statement

This study was approved by the Ethics Committee for Human Research, Central South University and was carried out in accordance with Declaration of Helsinki.

## Author Contributions

ZW, RL, and YZ carried out studies and wrote the manuscript. ZY, XM, and RL designed the study and revised the manuscript. ZY and XM performed the statistical analysis. DL, YY, and QZ collected specimens and experimental materials. All authors read and approved the final manuscript.

## Conflict of Interest

The authors declare that the research was conducted in the absence of any commercial or financial relationships that could be construed as a potential conflict of interest.
